# Alveolar Hemorrhage, a Rare and Life-Threatening Complication of Catastrophic Antiphospholipid Syndrome

**DOI:** 10.1155/2019/3284258

**Published:** 2019-11-13

**Authors:** Grace Loza, Carlos Hallo, Byron Chiliquinga, Alejandro Hallo

**Affiliations:** ^1^Internal Medicine, Eugenio Espejo Hospital, Quito, Ecuador; ^2^Internal Medicine, NYU-Winthrop Hospital, New York, NY, USA

## Abstract

Alveolar hemorrhage is the rarest pulmonary complication of catastrophic antiphospholipid syndrome and is associated with high mortality risk. This life-threatening complication results from autoimmune damage to the alveolar blood vessels. Given the limited literature addressing the association of these two pathologies, we report a series of three cases with this complication and then compare our findings with 6 cases reported in the literature.

## 1. Introduction

Antiphospholipid syndrome (APS) is a disorder that leads to a state of hypercoagulability characterized by antibodies against phospholipids resulting in thrombosis of veins or arteries. These antibodies were first described in 1906 in serological patients positive for syphilis [1].

Catastrophic antiphospholipid syndrome (CAPS) is a rare complication of APS occurring in approximately 1% of patients with a higher prevalence in women. APS is characterized by microvascular thrombosis producing multiorganic failure, the kidney being the most commonly affected organ, followed by the lungs and the central nervous system (CNS). In patients with pulmonary involvement, only 6–10% have alveolar hemorrhage which is commonly associated with microangiopathic hemolytic anemia and thrombocytopenia [2].

Certain clinical conditions may predispose to the development of CAPS including infections, malignancies, surgeries, and subtherapeutic anticoagulation. Several sets of diagnostic criteria have been proposed to stratify this rare pathology with high mortality risk. In our patients, we used the diagnostic criteria described by Asherson et al. and reviewed by Cervera et al. due to its high positive and negative predictive value. Cervera et al. acknowledge the difficulty to use biopsy to confirm occlusive microthrombi in patients in critical condition and suggest clinical and laboratory criteria to be used instead as described in Table 1. To assess and confirm thrombotic microangiopathy (TMA) in organs, we used the diagnostic workup used by Cervera et al. (Table 2) [2, 3].

The thrombotic events of this disease can occur in any blood vessel producing a wide variety of manifestations. The most common types of manifestation are associated with peripheral thrombosis (deep venous thrombosis in 38.9% of cases) and neurology (migraine 20.2% of the cases) [4].

CAPS has also been associated with pulmonary manifestations; the most common ones are acute respiratory distress syndrome, chronic thrombotic pulmonary disease, and secondary pulmonary hypertension. These manifestations are due to the disruption of the vessels surface. Alveolar hemorrhage is the rarest complication of CAPS, and it is caused by the disruption in the alveolar capillaries, leading to bleeding in the alveolar space [5, 6]. Alveolar hemorrhage's mortality rate increases from 35% in the absence of CAPS to approximately 50% in association with CAPS even in patients under treatement [7]. The clinical presentation of alveolar hemorrhage can vary from asymptomatic patients to severe respiratory failures [8]. The most frequent symptoms are dyspnea (64%), hypoxemia (55%), and cough (41%). The nonspecific nature of these symptoms urges to consider three important components in the diagnosis as the presence of blood in low respiratory tract including bloody secretion (57%), hemoptysis, or bloody secretions evidenced during tracheal aspirate or bronchoscopy, hemoglobin level below 1.5–2 g/dL with no evidence of bleeding in any other organ and no evidence of hemolysis, and diffuse infiltrate pattern in chest X-ray [8, 9].

## 2. Case 1

A 37-year-old female patient with APS diagnosed 17 years ago is brought to the emergency department due to syncope. The past medical history was positive for multiple transfusions due to hemolytic anemia and decreased visual acuity in recent months accompanied by continuous headaches of moderate intensity.

Laboratory tests showed anticardiolipin antibodies (ACA) IgG of 120 GPL-U/ml (positive: >10 GPL-U/ml) and IgM of 6 MPL-U/ml (negative: <7 MPL-U/ml). Severe microcytic anemia (HB: 3.6 g/dl) and thrombocytopenia require transfusion of two globular concentrates and five units of platelets. Peripheral blood smear revealed schistocytes.

Immunological tests were requested due to the suggestive autoimmune anemia, which showed positive anti-nuclear antibody, anti-RO antibody of 17 IU/ml (15–25 IU/ml), C3 of 0.92 g/L (positive >0.90 g/L), C4 of 0.21 g/L (negative <0.10 g/L), anti-thyroglobulin antibody 19.5 IU/ml (negative: <100 IU/ml), and incompatibility for all blood groups.

**Figure 1:**
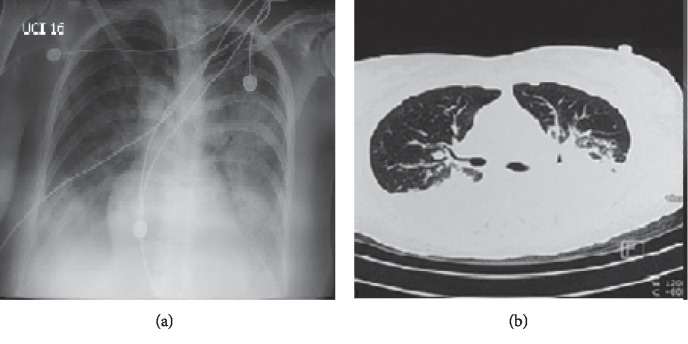
(a) Chest X-ray; AP view: bilateral, diffuse basal and perihilar alveolar infiltrate; (b) chest CT: bilateral effusion, increased vascularity, with bilateral infiltrates that converges to the posterior area.

The patient was admitted to MICU and was placed on medium-dose corticosteroids, prophylactic anticoagulation with low-molecular weight heparins, and additional globular concentrates transfusions. Anemia was corrected with the treatment; however, the patient's condition deteriorated due to severe respiratory distress, tachypnea, hypoxia, and SO_2_ 76%. A chest X-ray and chest CT scan showed bilateral, diffuse alveolar infiltrate (Figure 1).

Two major components of alveolar hemorrhage were evidenced in this patient: macrophages loaded with hemosiderin with macroscopic bleeding were found in bronchoscopy and severe thrombocytopenia. Despite the presence of alveolar hemorrhage, no hemoptysis was observed. Low-weight heparin was switched to unfractionated heparin, despite severe platelet disease.

Dialysis supplementation was started due to severe renal failure.

In less than seven days from the respiratory failure, the patient complained of persistent headaches. Further investigation revealed the occurrence of cerebral venous sinus thrombosis.

The patient met the criteria for definite CAPS. For these reasons, the patient was placed on enoxaparin 60 mg every 12 hours SubQ, prednisone 60 mg every 12 hours PO, and mycophenolate 2 g daily.

The patient improved and was discharged on mycophenolate and warfarin to a target INR of 3 with follow-up appointments in the outpatient clinic twelve weeks after discharge; anti-phospholipid antibody testing was repeated and confirmed the diagnosis of APS. Of note, a renal biopsy with immunofluorescence was also performed and ruled out lupus nephritis as a possible cause of renal failure.

## 3. Case 2

A 42-year-old female with a past medical history of rheumatoid arthritis diagnosed three months prior to admission, managed with methotrexate with poor adherence, was admitted due to psychomotor agitation without focal neurological deficits and fever (100.4°F) with neutrophilia.

Lumbar puncture showed a low glucose level in the cerebrospinal fluid (CSF) (glucose CSF 24.8 mg/dl, glucose serum: 78 mg/dl), 23 common germs in FilmArray (multiplex PCR system), as well as Gram and Chinese ink negative.

The patient lived in a tuberculosis endemic zone. Antibiotic therapy, antituberculosis drugs, and antifungal agents were initiated despite negative results.

The patient showed a sudden improvement in consciousness three days after starting treatment. However, she presented a new episode of altered mental status two days later, and it was associated with transaminases elevation 100 times above the reference value. Liver enzymes remained elevated despite discontinuation of antituberculosis agents. Brain CT scan of the brain showed ischemic areas in the corpus callosum and bilateral parietal lobes.

One day later, our patient presented severe hemodynamic decompensation with metabolic acidosis, acute respiratory failure, and pancytopenia. The patient was transferred to the MICU to provide mechanical ventilation and vasopressors.

Simultaneously, a progressive drop in hemoglobin was identified, so a bronchial tract lavage (not alveolar) was performed obtaining bloody secretions. Chest X-ray showed diffuse bilateral infiltrates. Patient's critical condition disabled him to undergo chest CT scan. Autoimmune markers were requested due to the suspicion of immune alveolar hemorrhage showing anti-cardiolipin antibodies IgM 0.3 MPL-U/ml (negative: <7 MPL-U/ml), IgG 100 MPL-U/ml (POSITIVE: >17 GPL-U/ml), Anti-DNA 29 IU/ml (positive: >20 IU/ml), and both positive anti-nuclear antibodies and lupus anticoagulant. Schistocytes were identified in peripheral blood smear. There was no evidence of lupus nephritis.

The patient met the criteria for definite CAPS associated with alveolar hemorrhage. Intravenous pulses of methylprednisolone 1 g daily IV and enoxaparin 60 mg daily SubQ to maintain isocoagulation state due to risk of hemorrhagic conversion of ischemic strokes were added to the already placed ventilatory support. The patient's critical condition prevented her from underwent bronchoscopy or pulmonary biopsy.

Despite the initial clinical improvement, the patient died in later days due to a new deterioration of respiratory function, renal failure, and encephalopathy.

## 4. Case 3

A 40 year-old male patient with a past medical history of hypertension, left nephrectomy 7 years ago due to renal artery thrombosis, ischemic cerebrovascular disease, and chronic kidney disease under clinical treatment, presented to the Emergency Department complaining of sudden hemoptysis, fatigue, and dyspnea of small efforts.

In the Emergency Department, physical examination showed altered mental status with neurological deficits, crackles in lung bases bilaterally, cyanosis, and 60% oxygen saturation without supplemental oxygen.

Additionally, metabolic acidosis (pH: 7.31, pCO_2_: 25.7 mmHg, pO_2_: 31 mmHg, HCO_3_: 12.7 mmol/L, EB: 36.4), severe normochromic normocytic anemia (hemoglobin: 7.72 gr/dl, hematocrit: 22.02%), and thrombocytopenia (5000 platelets) were observed with no evidence of bleeding. Schistocytes were identified in peripheral blood smear.

**Figure 2:**
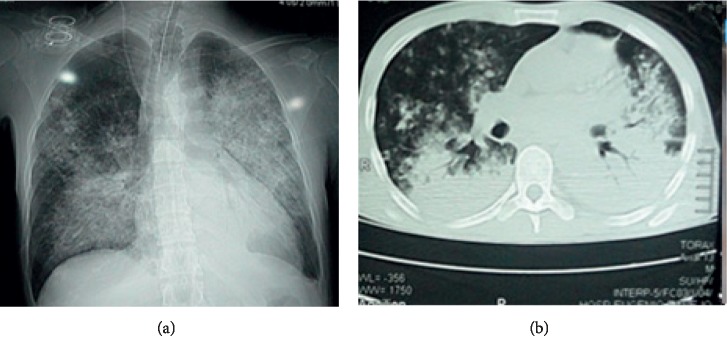
(a) Chest X-ray; AP view: diffuse infiltrate redistributed to the bases; (b) chest CT: diffuse bilateral alveolar infiltrate.

**Figure 3:**
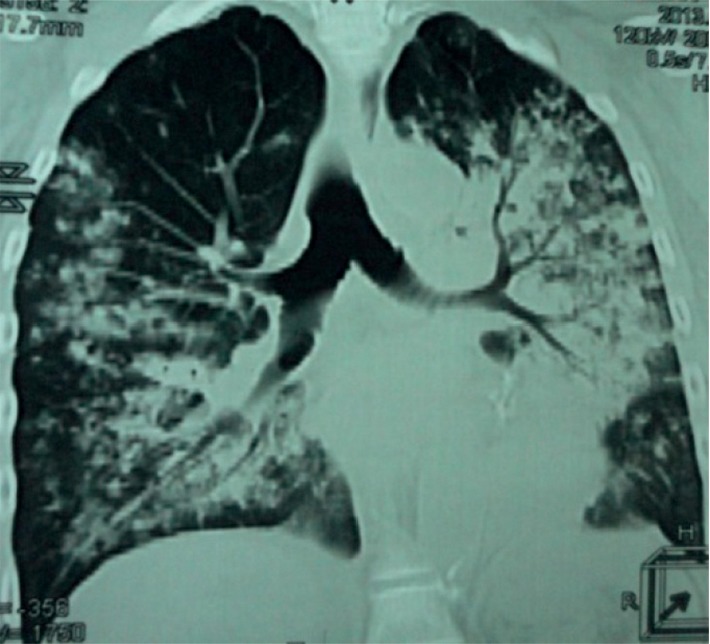
Chest CT: bilateral alveolar infiltrates in a ground-glass pattern.

A chest X-ray and CT scan were performed, showing diffuse bilateral alveolar infiltrate with perihilar and basal predominance and no important involvement of apexes or periphery (Figure 2) and bilateral alveolar infiltrates in a ground-glass pattern (Figure 3). Brain CT scan showed signs of ischemic areas in the brain parenchyma.

The patient was diagnosed with diffuse alveolar hemorrhage, and further investigation of immunological markers was ordered to identify the etiology. Anti-cardiolipin antibodies IgM 0.9 MPL-U/ml (negative: <7 MPL-U/ml), IgG 155 GPL-U/ml (positive: >17 GPL-U/ml), ANCA-C, and ANCA-P were negative, and anti-nuclear antibodies were positive. There was no evidence of lupus nephritis.

The patient was transferred to MICU due to hemodynamic instability and need for ventilatory support. It was not possible to perform lung biopsy or bronchoalveolar lavage due to the patient's critical condition.

The patient met the criteria for probable CAPS. Enoxaparin 60 mg every 12 hours SubQ and pulses of methylprednisolone 1 g daily IV were administered. Plasmapheresis was performed with no signs of clinical improvement; therefore, the patient was switched to immunoglobulins IV showing remarkable improvement. After overcoming the critical period, the patient was kept on chronic low-molecular weight heparin anticoagulant treatment. The patient was discharged on prednisone 30 mg daily and warfarin to a target INR of 3. Twelve weeks after discharge, anti-phospholipid antibody testing was repeated and the diagnosis of APS was confirmed.

## 5. Discussion

We present three diagnoses of diffuse alveolar hemorrhage in patients with CAPS with nonspecific clinical presentation. In all cases, the clinical presentation was sudden and unexpected and deteriorated rapidly within a one-week period. Out of the three patients, two fully recovered while one deceased. Given the limited literature addressing the association of alveolar hemorrhage and CAPS, we compared these 3 cases treated in our hospital with cases reported in the literature (Tables 3 and 4).

The initial respiratory symptoms our patients were cough, respiratory distress, and oxygen saturation less than 85% without oxygen support and hemoptysis. Those findings are consistent with the cases reported in the literature [9]. One of our patients presented hemoptysis, a finding listed in a third of patients with alveolar hemorrhage [5, 10].

Encephalopathy is a complication in 40.2% of CAPS cases. Even though our second patient was diagnosed with meningitis due to the presence of fever and altered mental status, it is possible that the patient's clinical presentation was a complication from CAPS itself rather than an infectious condition because of negative CSF FilmArray report. We were unable to find cases with similar initial presentations in the literature.

Both radiographic and tomographic findings were consistent with those found in the literature, diffuse areas of ground-glass opacities or consolidations in chest X-rays and infiltrated patches in the tomography. In three of the reported cases, the findings were bilateral.

Alveolar hemorrhage in APS can be explained due to anti-phospholipid antibodies binding to the alveolar surface [6]. Such antibodies can be found in both asymptomatic patients and patients with nonthrombotic manifestations. Thrombotic manifestations occur in half of the patients and hemolytic anemia in one-third of patients [9, 10].

The thrombotic effect can be explained by the inhibition of anticoagulant systems, preventing the degradation of factor V and VII altering the protein C system and the inhibition of fibrinolysis [11]. Most antibodies bind to phospholipid-binding proteins expressed on cell surfaces, endothelium, and platelets, which half-explain their pathogenesis [12]. The activation of the endothelial surface transforms the vessel wall into a procoagulant surface [9, 13].

Lupus anticoagulant was positive in seven out of nine patients being the most common finding. Anti-cardiolipin antibodies were present in five out of nine patients. Anti-*β*2glycoprotein (Anti-*β*2GPI) antibodies were positive in only four of the patients [14–17]. No changes were reported in prothrombin.

The initial management of alveolar hemorrhage is ventilatory support and anticoagulation, followed by the treatment of CAPS. Anticoagulation is key in the treatment since it inhibits clot formation and promotes fibrinolysis and could also have an effect on preventing anti-phospholipid antibodies from binding to the endothelial surface [6]. Heparin use is recommended at doses that do not increase the risk of bleeding; however, there is insufficient evidence to support a dose that avoids this risk. After anticoagulation, the use of high-dose corticosteroids is necessary to act on mediators involved in CAPS. The use of cyclophosphamide is reserved for those patients with autoimmune diseases. Plasmapheresis could have a beneficial effect by removing free antibodies, as well as other mediators responsible for the pathophysiology of catastrophic APC [6, 10, 12].

## Figures and Tables

**Table 1:** Preliminary criteria for the classification of catastrophic antiphospholipid syndrome (CAPS) [3].

(1) Evidence of involvement of three or more organs, systems, and/or tissues^a^
(2) Development of manifestations simultaneously or in less than one week
(3) Confirmation by histopathology of small vessel occlusion in at least one organ or tissue^b^
(4) Laboratory confirmation of the presence of anti-phospholipid antibodies (lupus anti-coagulant and/or anti-cardiolipin antibodies)^c^
Definite CAPS:
(i) All four criteria
Probable CAPS
(i) All four criteria, except for only two organs, systems, and/or tissues involved
(ii) All four criteria, except for the absence of laboratory confirmation owing to the early death of a patient never tested for anti-phospholipid antibodies before the CAPS
(iii) Criteria (1), (2), and (4)
(iv) Criteria (1), (3), and (4) and the development of a third event between one week and one month after presentation, despite anticoagulation

Note: given that many times biopsy and histological confirmation of small vessel occlusion cannot be obtained due to the critical condition of the patients, a proposal has been made to substitute the “histopathology criteria” by the exclusion of other diagnoses. ^a^Usually clinical evidence of vessel occlusions, confirmed by imaging techniques when appropriate; renal involvement is defined by a 50% rise in serum creatinine, severe systemic hypertension (>180/100 mm Hg), and/or proteinuria (>500 mg/24 h). ^b^For histopathological confirmation, significant evidence of thrombosis must be present, although vasculitis may coexist occasionally. ^c^If the patient had not previously been diagnosed as having an APS, the laboratory confirmation requires that the presence of anti-phospholipid antibodies must be detected on two or more occasions at least 12 weeks apart (not necessarily at the time of the event), according to the proposed preliminary criteria for the classification of definite APS.

**Table 2:** Diagnostic workup in front of a patient with suspicion of thrombotic microangiopathy (TMA) [3].

*(1) To establish the suspicion of TMA*
(i) Thrombocytopenia (<150 × 109/l or >25% of decrease)
(ii) Signs of microangiopathic hemolysis
(iii) Anemia (±increase in mean corpuscular volume)
(iv) Reticulocyte count raised
(v) Lactate dehydrogenase (LDH) increased with haptoglobin decreased
(vi) Direct Coomb's test negative
(vii) Blood smear searching schistocytes

*(2) To look for organ involvement*
(i) Neurological: confusion, headache, seizures, encephalopathy, and focal deficits
(ii) Renal: ARF, arterial hypertension, proteinuria, and hematuria
(iii) Cardiac: cardiac failure, hypotension, and ischemic cardiopathy
(iv) Pulmonary: ARDS and respiratory insufficiency
(v) Gastrointestinal: abdominal pain, intestinal angina, diarrhea, and vomiting
(vi) Hematological (thrombocytopenia): epistaxis, hemoptysis, menorrhagia, retinal hemorrhage, gastrointestinal bleeding, and petechiae

*(3) To confirm organ involvement*
(i) Blood analysis including renal function, cellular blood count, LDH, liver and pancreatic enzymes, creatin kinase, and troponin I
(ii) Renal biopsy: to confirm glomerular microthrombosis
(iii) CT/MRI brain: to determine neurological involvement
(iv) Electrocardiogram/echocardiogram: to document or monitor cardiac damage
(v) Chest radiograph/CT: to document lung involvement
(vi) Echography/CT: to document hepatic/pancreatic/intestinal involvement
(vii) Fundoscopic examination: to document retinal vessel involvement

*(4) To investigate the etiology*
(i) ADAMTS 13 activity: <5–10% (TTP)
(ii) If gastroenteritis (bloody diarrhea): Shiga toxin/STEC: positive (HUS)
(iii) If ADAMTS13 > 10%: secondary or associated TMA
(iv) Fundoscopic examination (malignant hypertension)
(v) Immunologic profile: ANA, ANCA, and aPL (autoimmune diseases)
(vi) Pregnancy test (pregnancy-related)
(vii) CT thoracoabdominal or PET: cancer-associated
(viii) Clinical history looking for drugs/heparin and anti-PF4 antibodies (HIT)
(ix) Complement studies: FH, FB, FI, anti-FH antibodies, and genetic study (aHUS)

aHUS: atypical HUS, ANA: anti-nuclear antibodies, ANCA: anti-neutrophil cytoplasmic antibodies, aPL: anti-phospholipid antibodies, CT: computed tomography, HIT: heparin-induced thrombocytopenia; HUS: hemolytic uremic syndrome, PET: positron emission tomography, STEC: Shiga toxin *Escherichia coli*, TTP: thrombotic thrombocytopenic purpura.

**Table 3:** Comparison of characteristics of patients with alveolar hemorrhage.

Case reports	Clinical presentation	Physical exam	Chest X-ray	CT	Laboratory	Lupus anticoagulant	ANA	ANCA
Case 1	Decreased visual acuity syncope cough	Lung crackles	Diffuse alveolar infiltrate	No major changes	Microcytic anemia, thrombocytopenia	Positive	Positive	Positive

Case 2	Alteration of the state of consciousness cough respiratory distress	Lung crackles	Diffuse alveolar infiltrate	Ischemic areas in the corpus callosum and parietal lobe	Pancytopenia	Not requested	Positive	Negative

Case 3	Hemoptysis dyspnea	Lung crackles	Ground glass	Diffuse alveolar infiltrate	Normocytic normochromic anemia	Positive	Positive	Negative

Rangel et al. [14]	Abdominal pain Diarrhea Dyspnea	Lung crackles	Patched opacities in middle and basal lobes	Ground glass Hepatosplenomegaly	Abnormal liver function Normochromic normocytic anemia High LDH Hypokalemia Hypoalbuminemia	Positive	Positive	Negative

Wan and Tsang [18]	Asymptomatic	Venous thromboembolism	Opacities	Bilateral patched consolidations with consolidations and interlobular septal thickening	Thrombocytopenia	Positive	Not reported	Not reported

Isshiki et al. [19]	Hemoptysis Fever Dyspnea	Left lung crackles	Diffuse infiltrates in the left lung	Ground glass pulmonary artery thrombosis in the right basal lobe	Not reported	Positive	Negative	Negative

Martis et al. [15]	Respiratory distress	Not reported	Not reported	Not reported	C4 complement fraction decreased	Positive	Not reported	Negative

Hambly et al. [16]	Dyspnea hemoptysis	Not reported	Abnormal pattern not specified	Ground glass	Thrombocytopenia, lymphopenia	Not reported	Not reported	Negative

Nguyen et al. [17]	Dyspnea Productive cough hemoptysis	Not reported	Small patched infiltrate	Infiltrate patched in the right middle lobe	Thrombocytopenia	Positive	Positive	Not reported

**Table 4:** Comparison of characteristics of patients with alveolar hemorrhage.

Prothrombin	ACA IgM/IgG	Anti-*β*2GPI	Heparin	Treatment	Evolution	Bronchoalveolar lavage	Bronchoscopy
Normal	IgM negative IgG 120 GPL/ml	Negative	Enoxaparin	Prednisone 60 mg PO QD mycophenolate	Dyspnea of medium efforts	Macrophages loaded with hemosiderin	Not ordered

Normal	IgM negative IgG 100 GPL/ml	Negative	Enoxaparin	Methylprednisolone 1 gr IV QD	Died	Not ordered	Not ordered

Normal	IgM negative IgG 155 GPL- U/ml	No ordered	Enoxaparin	Methylprednisolone, immunoglobulins	Clinical improvement	Not ordered	Not ordered

Normal	Not reported	Positive	Not initiated	Methylprednisolone 1 g QD; fresh frozen plasma three units	Clinical improvement	Bloody	Friable mucosa with macroscopic blood

Not reported	Positive (unreported values)	Negative	Discontinued due to surgery	Anticoagulants, steroids, and plasmapheresis	Infarction in a left pulmonary lobe	Bloody	Diffuse submucosal bleeding

Normal	Not reported	Negative	Not reported	Methylprednisolone 1 g QD; prednisone 25 mg QD	Dyspnea recurrence and hemoptysis	Bloody	Not reported

Not reported	Positive	Positive	Discounted due to bleeding	Methylprednisolone 1 g QD; rituximab 375 mg/m^2^	Clinical improvement	Not reported	Massive alveolar hemorrhage

Not reported	Positive	Positive	Not reported	High doses of corticosteroids, cyclophosphamide	Image improvement	Macrophages loaded with hemosiderin	Not reported

Not reported	Negative	Positive	Not reported	Oral methylprednisolone, aspirin	Hemoptysis and dyspnea recurrence	Bloody	Bloody
